# Exploring genetic associations of Crohn’s disease and ulcerative colitis with extraintestinal cancers in European and East Asian populations

**DOI:** 10.3389/fimmu.2024.1339207

**Published:** 2024-02-08

**Authors:** Chengdong Yu, Jiawei Xu, Siyi Xu, Lei Tang, Qinyuan Han, Xiaoqiang Zeng, Yanxiao Huang, Tenghua Yu, Zhengkui Sun

**Affiliations:** ^1^ Jiangxi Medical College, Nanchang University, Nanchang, China; ^2^ Department of breast surgery, Jiangxi Cancer Hospital, Nanchang, China

**Keywords:** Crohn’s disease, ulcerative colitis, extraintestinal cancer, mendelian randomization, genetic association

## Abstract

**Background:**

Previous studies have reported associations of Crohn’s disease (CD) and ulcerative colitis (UC) with the risks of extraintestinal cancers, but the causality remains unclear.

**Methods:**

Using genetic variations robustly associated with CD and UC extracted from genome-wide association studies (GWAS) as instrumental variables. Nine types of extraintestinal cancers of European and Asian populations were selected as outcomes. We used the inverse variance weighted method as the primary approach for two-sample Mendelian randomization analysis. Sensitivity analyses were carried out to evaluate the reliability of our findings.

**Results:**

In the European population, we found that CD showed a potential causal relationship with pancreatic cancer (OR: 1.1042; 95% CI: 1.0087-1.2088; P=0.0318). Meanwhile, both CD (outliers excluded: OR: 1.0208; 95% CI: 1.0079-1.0339; P=0.0015) and UC (outliers excluded: OR: 1.0220; 95% CI: 1.0051-1.0393; P=0.0108) were associated with a slight increase in breast cancer risk. Additionally, UC exhibited a potential causal effect on cervical cancer (outliers excluded: OR: 1.1091; 95% CI: 1.0286-1.1960; P=0.0071). In the East Asian population, CD had significant causal effects on pancreatic cancer (OR: 1.1876; 95% CI: 1.0741-1.3132; P=0.0008) and breast cancer (outliers excluded: OR: 0.9452; 95% CI: 0.9096-0.9822; P=0.0040). For UC, it exhibited significant causal associations with gastric cancer (OR: 1.1240; 95% CI: 1.0624-1.1891; P=4.7359×10^–5^), bile duct cancer (OR: 1.3107; 95% CI: 1.0983-1.5641; P=0.0027), hepatocellular carcinoma (OR: 1.2365; 95% CI: 1.1235-1.3608; P=1.4007×10^–5^) and cervical cancer (OR: 1.3941; 95% CI: 1.1708-1.6599; P=0.0002), as well as a potential causal effect on lung cancer (outliers excluded: OR: 1.1313; 95% CI: 1.0280-1.2449; P=0.0116).

**Conclusions:**

Our study provided evidence that genetically predicted CD may be a risk factor for pancreatic and breast cancers in the European population, and for pancreatic cancer in the East Asian population. Regarding UC, it may be a risk factor for cervical and breast cancers in Europeans, and for gastric, bile duct, hepatocellular, lung, and cervical cancers in East Asians. Therefore, patients with CD and UC need to emphasize screening and prevention of site-specific extraintestinal cancers.

## 1 Introduction

Crohn’s disease (CD) and ulcerative colitis (UC) are the main subtypes of inflammatory bowel disease (IBD), which are chronic inflammatory disorders that primarily affect the gastrointestinal tract (1–3). It is characterized by periods of remission and flare-ups, leading to impaired quality of life for patients and substantial costs for health care (4, 5).

The risk of intestinal cancer in CD and UC has been analyzed deeply (6, 7), but the potential association of CD and UC with extraintestinal cancer has received relatively little attention. However, extraintestinal manifestations are observed in up to 35% of IBD patients (8, 9), highlighting the importance of studying the risk of extraintestinal cancer in this population.

Several observational studies have shown that IBD patients were positively associated with the risk of extraintestinal cancer. A meta-analysis of population-based cohort studies, involving 17,052 IBD patients, indicated that CD patients exhibited increased risks of cancer in the upper gastrointestinal tract, lung, and skin, while UC patients had a higher risk of liver-biliary cancer (10). Another meta-analysis suggested a significant association between IBD, especially UC, and an increased risk of cervical cancer (11). Moreover, a 20-year prospective follow-up study in Norway has reported an increased incidence of breast cancer in patients with CD and UC (12). Results from some cohort and case-control studies have reported an elevated risk of pancreatic cancer and skin cancer in patients with IBD (13–15). However, there are currently no specific guidelines for extraintestinal cancer screening or surveillance in patients with CD and UC. Additionally, it is not easy to assess the real risk and causality of extraintestinal cancer in CD and UC due to the residual confounding commonly encountered in traditional observational studies.

Mendelian randomization (MR) is a new method of etiological investigation that uses genetic variations closely linked to a particular exposure as instrumental variables (IVs) (16). This approach can avoid the limitations of traditional epidemiological studies and permit us to draw causal inferences about the impact of specific exposures on outcomes (17). Because alleles follow the principle of random allocation during gametogenesis, the offspring have random genetic variations; thus, the results are not affected by confounding factors or reverse causation (18, 19).

Our study aimed to appraise causal associations of CD and UC with extraintestinal cancers through MR analysis. Furthermore, we tried to analyze the differences between European and East Asian ethnic groups. Significantly, our findings have the potential to inform more effective and targeted cancer surveillance programs for patients with CD and UC, facilitating early cancer detection and alleviating the burden on healthcare systems.

## 2 Materials and methods

### 2.1 Study design

**Figure 1:**
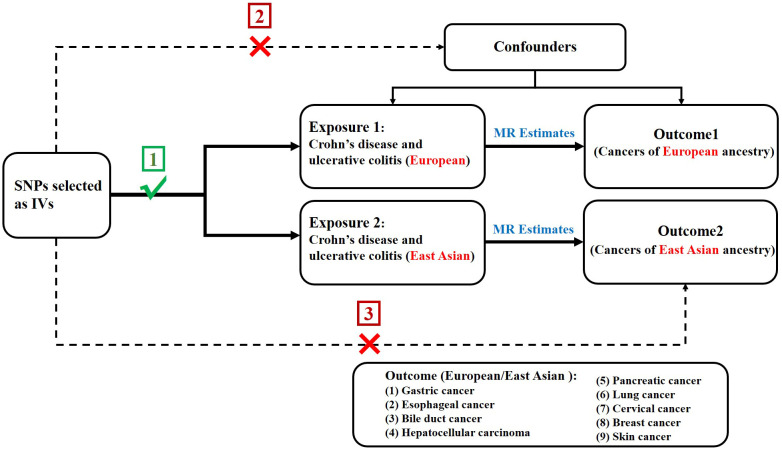
Design diagram of the MR analysis.

In order to assess the causal association of CD and UC with extraintestinal cancer, we conducted a two-sample MR study. The single nucleotide polymorphisms (SNPs) selected as IVs were required to meet three following key premises (20) (1): SNPs must be intensely linked to exposure; (2) SNPs must not be linked to confounding factors; and (3) SNPs should not be directly linked to outcome (**Figure 1**).

### 2.2 Data source

The summary genome-wide association study (GWAS) data for CD (Europeans: 17,897 cases/33,977 controls; East Asians: 1,690 cases/3,719 controls) and UC (Europeans: 13,768 cases/33977 controls; East Asians: 1,134 cases/3,719 controls) were obtained from the International Inflammatory Bowel Disease Genetics Consortium (IIBDGC) (21). And the summary GWAS data for extraintestinal cancers included in this study were extracted directly or indirectly from the IEU Open GWAS project (https://gwas.mrcieu.ac.uk/). See **Tables 1**, **2** for more information about exposures and outcomes.

**Table 1:** Information on the datasets for exposures.

Exposure	ncase	ncontrol	Sample size	Consortium	Ancestry
Crohn’s disease	17,897	33,977	51,874	IIBDGC	European
Ulcerative colitis	13,768	33,977	47,745	IIBDGC	European
Crohn’s disease	1,690	3,719	5,409	IIBDGC	East Asian
Ulcerative colitis	1,134	3,719	4,853	IIBDGC	East Asian

**Table 2:** Information on the datasets for outcomes.

Outcome	European			East Asian		
	ncase/ncontrol	Data source	PMID	ncase/ncontrol	Data source	PMID
Gastric cancer	633/174,006	FinnGen	NA	6,563/195,745	BioBank Japan	32514122 (22)
Esophageal cancer	998/475,308	Sakaue S	34594039 (23)	1,300/195,745	BioBank Japan	32514122
Bile duct cancer	350/372,016	UK Biobank	NA	339/195,745	BioBank Japan	32514122
Hepatocellular carcinoma	168/372,016	UK Biobank	NA	1,866/195,745	BioBank Japan	32514122
Pancreatic cancer	1,896/1,939	PanScan1	19648918 (24)	442/195,745	BioBank Japan	32514122
Lung cancer	11,348/15,861	ILCCO	24880342 (25)	4,050/208,403	BioBank Japan	32514122
Cervical cancer	909/238,249	Sakaue S	34594039	605/89,731	BioBank Japan	32514122
Breast cancer	122,977/105,974	BCAC	29059683 (26)	5,552/89,731	BioBank Japan	32514122
Skin cancer	25,928/466,275	Sakaue S	34594039	154/178,572	Sakaue S	34594039

ILCCO, International Lung Cancer Consortium; BCAC, Breast Cancer Association Consortium.

### 2.3 SNP selection

**Figure 2:**
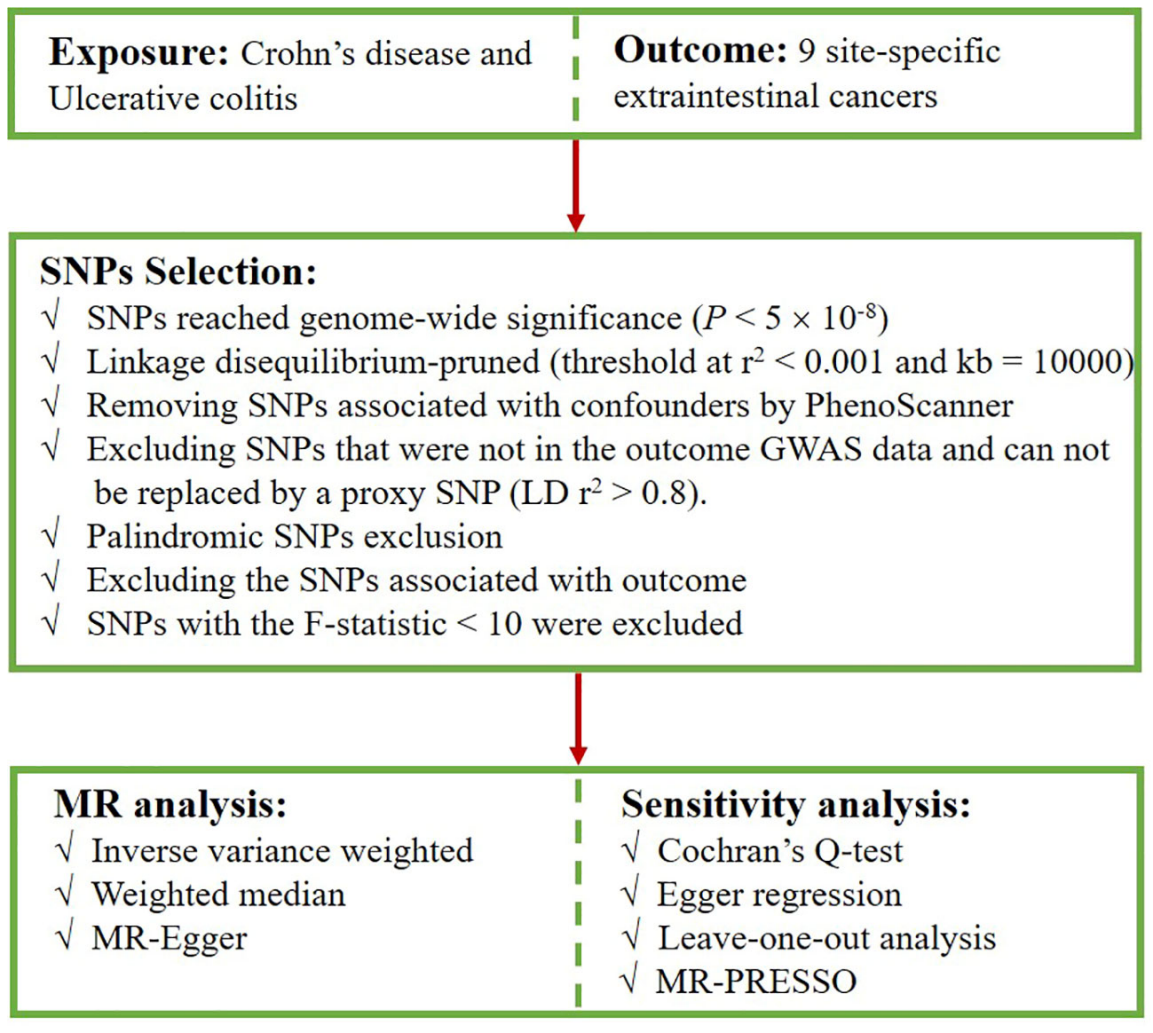
Flowsheet of SNPs selection in this study.

First, we extracted SNPs intensely associated with CD and UC from the corresponding datasets, with a screening condition of P < 5×10^-8^. Second, to ensure the independence of exposure instruments, SNPs with a low likelihood of linkage disequilibrium (LD) (r^2^ < 0.001, kb = 10,000) were retained (27). We checked the possible phenotypes of each SNP related to CD and UC at PhenoScanner (28), and SNPs directly linked to some recognized confounders associated with carcinogenesis were excluded, such as alcohol intake (29, 30), smoking (31), body-mass index (BMI) (32–34). The excluded SNPs and their relevant traits are presented in **Supplementary Table S1**. Subsequently, when the selected SNPs were not extractable from the outcome dataset, we opted for SNPs with strong correlations (r^2^ > 0.8) as proxies (35). Palindromic SNPs that may cause bias were removed. Furthermore, SNPs that were strongly correlated with the outcome were excluded because they deviated from the core assumption of the IVs. Finally, F-statistics were calculated (F = beta^2^/se^2^) to evaluate the potential for weak instrument bias, and any SNP with an F-statistic < 10 was excluded (36, 37). **Figure 2** presents the selection flowchart.

### 2.4 Statistical analysis

The inverse variance weighted (IVW) method is considered to be the most powerful method for detecting causation in MR analysis (38); therefore, the results were mainly based on the IVW method, supplemented by the weighted median and MR Egger. We used odds ratios (ORs) to express the effects of CD and UC on extraintestinal cancer risk. The presence of heterogeneity was determined using Cochran’s Q test, with P < 0.05 indicating heterogeneity (39). To detect pleiotropy, we used the MR-Egger regression test, whereby a non-zero intercept was indicative of horizontal pleiotropy (P < 0.05) (40). Moreover, we identified outliers by MR-PRESSO method and repeated MR analyses after excluding these outliers (41). Additionally, leave-one-out analysis was performed to assess the impact of a single SNP’s removal on the results (42).

To determine more rigorous causalities, we used a Bonferroni-corrected significance threshold calculated as 0.0056 (0.05/9, according to the 9 types of cancer). The P value between 0.0056 and 0.05 was considered to suggest a potential relationship between exposure and outcome.

This study followed the STROBE-MR guidelines (43). All analyses were conducted using the “TwoSampleMR” and “MRPRESSO” packages in R software (version 4.3.1).

## 3 Results

### 3.1 SNP Selection

In this study, we detected 122 SNPs for CD and 88 SNPs for UC in the European population, while in the East Asian population, we observed detections of 14 SNPs for CD and 10 SNPs for UC (**Supplementary Tables S2-S5**). The F-statistics of all SNPs were greater than 10, avoiding weak instrumental variable bias.

### 3.2 Analysis of the European population

**Figure 3:**
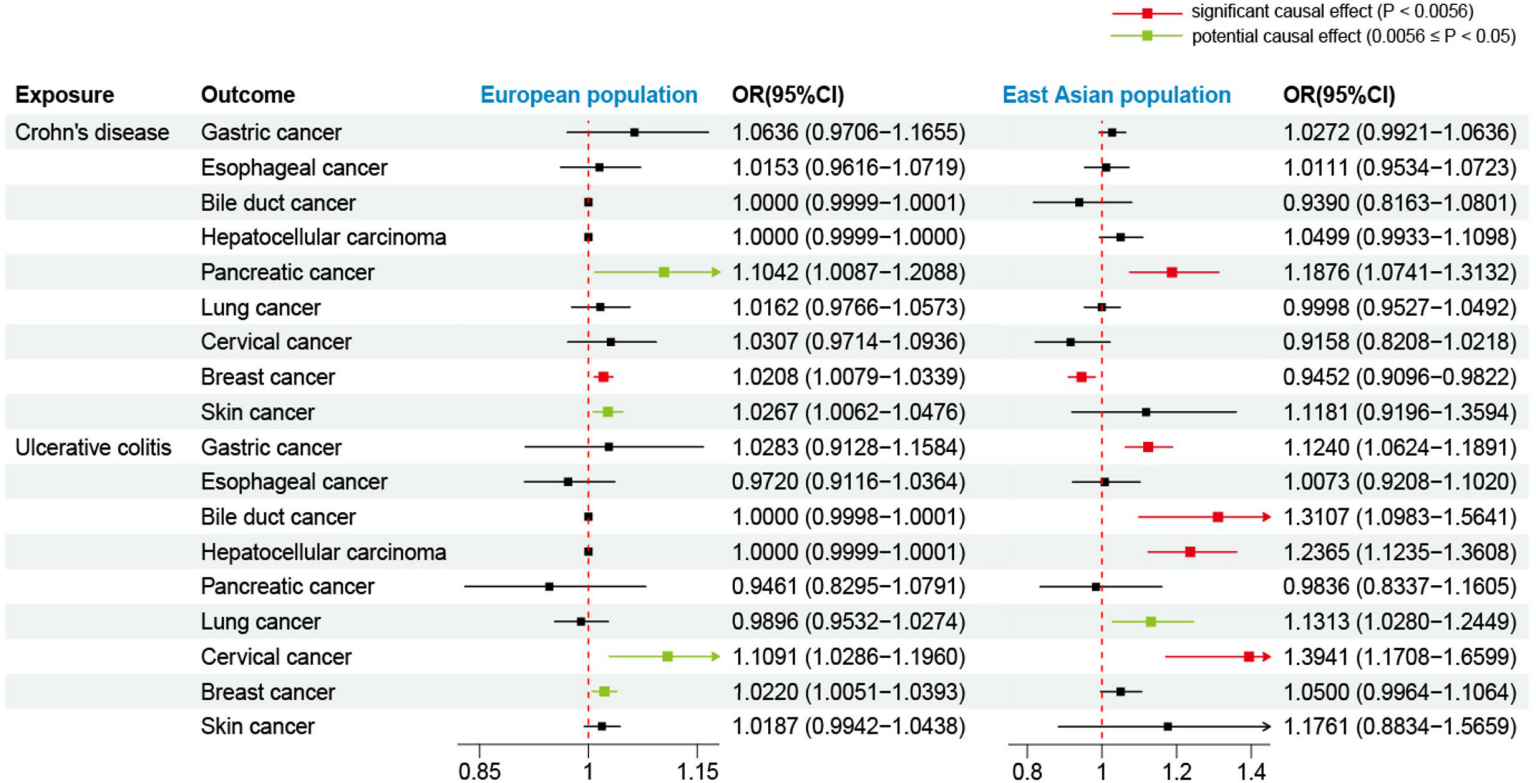
Forest plot for effects of Crohn’s disease and ulcerative colitis on the risk of extraintestinal cancers based on the IVW. The results show the ORs and CIs after excluding outliers. OR, odds ratio; CI, confidence interval.

**Figure 4:**
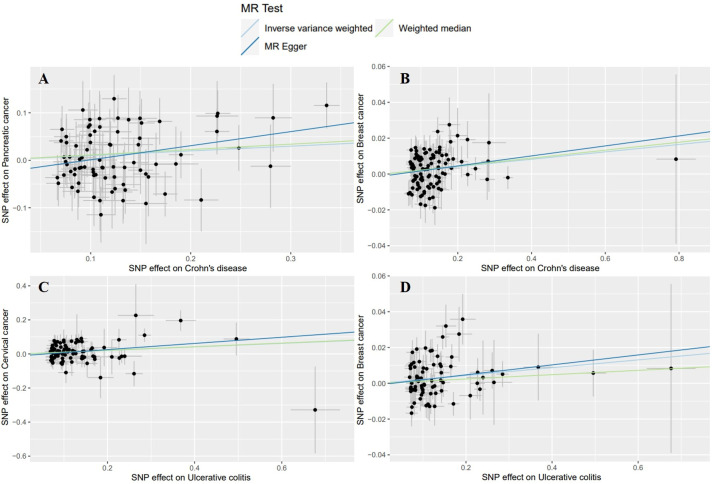
Scatter plots of the positive results for effects of Crohn’s disease and ulcerative colitis on the risk of extraintestinal cancers in the European population. **(A)** Crohn’s disease on pancreatic cancer **(B)** Crohn’s disease on breast cancer **(C)** ulcerative colitis on cervical cancer **(D)** ulcerative colitis on breast cancer.

Initially, we used the GWAS data of the European population to evaluate the effects of CD on extraintestinal cancer risk. Based on IVW analysis, we observed a potential association between CD per unit increase in logOR and pancreatic cancer (OR: 1.1042; 95% CI: 1.0087-1.2088; P=0.0318) as well as skin cancer (outliers excluded: OR: 1.0267; 95% CI: 1.0062-1.0476; P=0.0103). However, the MR-Egger method in skin cancer analysis showed OR<1 (**Supplementary Table S6**), which was inconsistent with the direction of IVW (OR>1); thus, we could not determine the causality of CD and skin cancer. In addition, CD was found to be significantly linked to breast cancer (outliers excluded: OR: 1.0208; 95% CI: 1.0079-1.0339; P=0.0015). Regarding UC, potentially positive correlations were found between UC and the risks of cervical cancer (outliers excluded: OR: 1.1091; 95% CI: 1.0286-1.1960; P=0.0071) and breast cancer (outliers excluded: OR: 1.0220; 95% CI: 1.0051-1.0393; P=0.0108) (**Table 3**, **Figure 3**). Scatter plots of the positive results illustrated causal estimates derived from each SNP (**Figure 4**). Sensitivity analyses showed that the effects of CD on pancreatic cancer and UC on cervical cancer were reliable, exhibiting no heterogeneity or horizontal pleiotropy. Although the impacts of CD and UC for breast cancer remained heterogeneity after removing outliers, there was no significant pleiotropy. Moreover, leave-one-out analysis demonstrated the reliability of the findings (**Supplementary Figure S1**). In addition, we discovered that CD and UC were not associated with the occurrence of the other extraintestinal cancers included in this study in the European population. More results of MR analyses are presented in **Supplementary Tables S6**, **S7**.

**Table 3:** Causal effects of Crohn’s disease and ulcerative colitis on the risk of extraintestinal cancers in the European population.

Exposure	Outcome	OR (95%CI)	P-_IVW_	P-_heterogeneity_	P-_pleiotropy_
Crohn’s disease	Gastric cancer	1.0636 (0.9706-1.1655)	0.1866	0.592	0.579
	Esophageal cancer	1.0153 (0.9616-1.0719)	0.5844	0.268	0.535
	Bile duct cancer	1.0000 (0.9999-1.0001)	0.9834	0.265	0.504
	Hepatocellular carcinoma	1.0000 (0.9999-1.0000)	0.4559	0.401	0.397
	Pancreatic cancer	**1.1042 (1.0087-1.2088)**	**0.0318**	0.370	0.067
	Lung cancer	1.0171 (0.9831-1.0523)	0.3274	0.009	0.824
	Cervical cancer	1.0307 (0.9714-1.0936)	0.3169	0.358	0.711
	Breast cancer	**1.0208(1.0079-1.0339)**	**0.0015**	0.005	0.659
	Skin cancer	**1.0267 (1.0062-1.0476)**	**0.0103**	1.304×10^–5^	0.270
Ulcerative colitis	Gastric cancer	1.0283 (0.9128-1.1584)	0.6463	0.207	0.409
	Esophageal cancer	0.9720 (0.9116-1.0364)	0.3851	0.390	0.172
	Bile duct cancer	1.0000 (0.9998-1.0001)	0.9131	0.138	0.806
	Hepatocellular carcinoma	1.0000 (0.9999-1.0001)	0.5928	0.450	0.605
	Pancreatic cancer	0.9461 (0.8295-1.0791)	0.4090	0.306	0.978
	Lung cancer	0.9896 (0.9532-1.0274)	0.5857	0.090	0.627
	Cervical cancer	**1.1091 (1.0286-1.1960)**	**0.0071**	0.179	0.348
	Breast cancer	**1.0220 (1.0051-1.0393)**	**0.0108**	0.0001	0.746
	Skin cancer	1.0187 (0.9942-1.0438)	0.1357	0.001	0.550

The results show the ORs and CIs after excluding outliers. IVW, inverse variance weighted; OR, odds ratio; CI, confidence interval. The bold values mean P-_IVW_<0.05.

### 3.3 Analysis of the East Asian population

**Figure 5:**
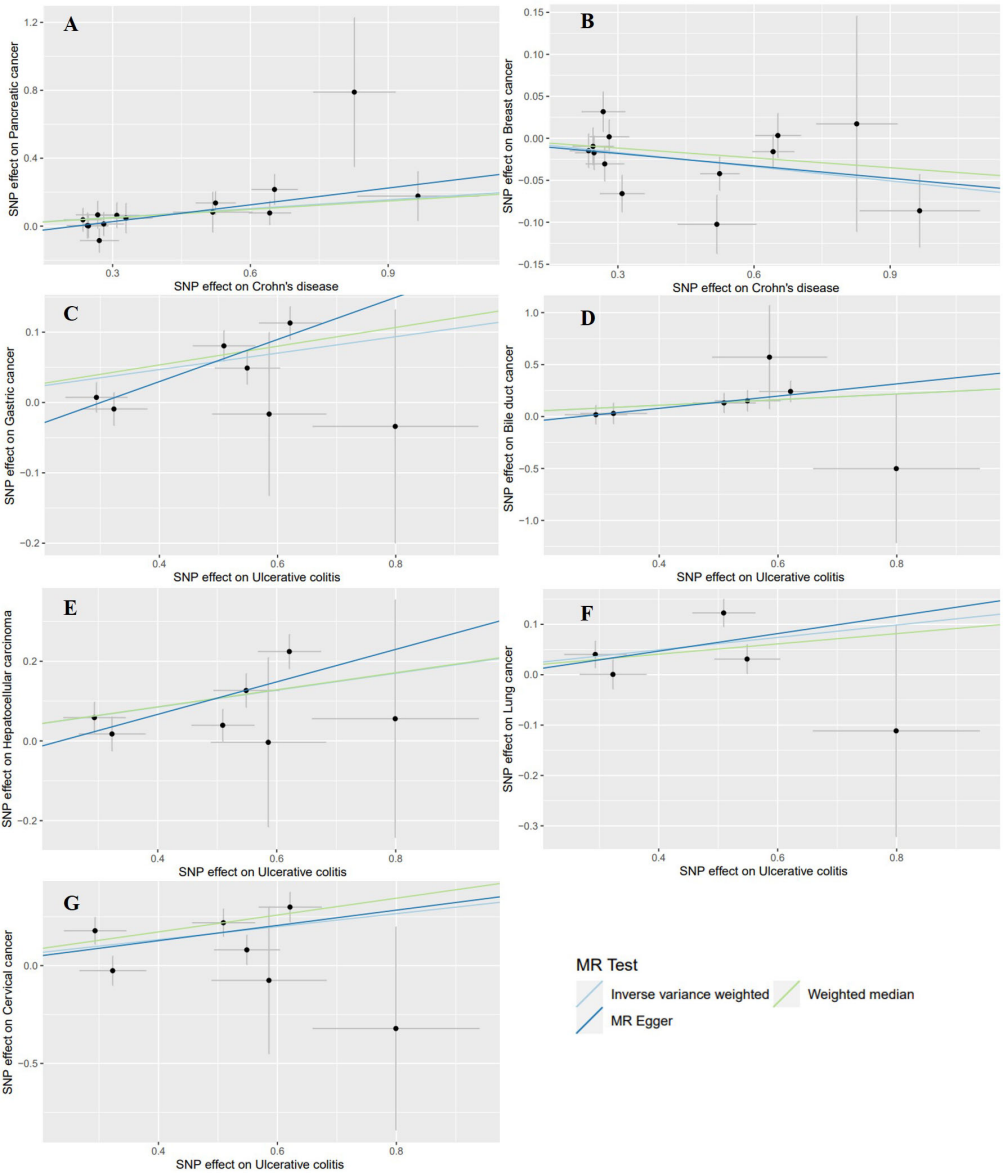
Scatter plots of the positive results for effects of Crohn’s disease and ulcerative colitis on the risk of extraintestinal cancers in the East Asian population. **(A)** Crohn’s disease on pancreatic cancer **(B)** Crohn’s disease on breast cancer **(C)** ulcerative colitis on gastric cancer **(D)** ulcerative colitis on bile duct cancer **(E)** ulcerative colitis on hepatocellular carcinoma **(F)** ulcerative colitis on lung cancer **(G)** ulcerative colitis on cervical cancer.

Using the GWAS data from the East Asian population, genetic prediction indicated a significant causal association between CD and an increased risk of pancreatic cancer (P=0.0008), with an OR of 1.1876 (95% CI: 1.0741-1.3132). Surprisingly, CD was found to be significantly negatively related to breast cancer risk (outliers excluded: OR: 0.9452; 95% CI: 0.9096-0.9822; P=0.0040). Regarding UC, we identified a significant causal effect between UC per unit increase in logOR and four types of extraintestinal cancer that are gastric cancer (OR: 1.1240; 95% CI: 1.0624-1.1891; P=4.7359×10^–5^), bile duct cancer (OR: 1.3107; 95% CI: 1.0983-1.5641; P=0.0027), hepatocellular carcinoma (OR: 1.2365; 95% CI: 1.1235-1.3608; P=1.4007×10^–5^), and cervical cancer (OR: 1.3941; 95% CI: 1.1708-1.6599; P=0.0002). At the genetic level, these findings indicate that UC increases the risk of developing the four types of cancer (**Table 4**, **Figure 3**). In addition, UC was found to be potentially positively associated with a higher risk of lung cancer (outliers excluded: OR: 1.1313; 95% CI: 1.0280-1.2449; P=0.0116). Scatter plots of the positive results illustrated causal estimates derived from each SNP (**Figure 5**). Sensitivity analyses showed that the above associations were robust in the East Asian population, displaying no significant heterogeneity or horizontal pleiotropy. Furthermore, leave-one-out analysis further illustrated the robustness of the results (**Supplementary Figure S2**). More results of MR analyses are presented in **Supplementary Tables S8**, **S9**.

**Table 4:** Causal effects of Crohn’s disease and ulcerative colitis on the risk of extraintestinal cancers in the East Asian population.

Exposure	Outcome	OR (95%CI)	P-_IVW_	P-_heterogeneity_	P-_pleiotropy_
Crohn’s disease	Gastric cancer	1.0272 (0.9921-1.0636)	0.1300	0.058	0.870
	Esophageal cancer	1.0111 (0.9534-1.0723)	0.7129	0.476	0.477
	Bile duct cancer	0.9390 (0.8163-1.0801)	0.3781	0.115	0.156
	Hepatocellular carcinoma	1.0499 (0.9933-1.1098)	0.0851	0.857	0.730
	Pancreatic cancer	**1.1876 (1.0741-1.3132)**	**0.0008**	0.781	0.182
	Lung cancer	0.9998 (0.9527-1.0492)	0.9927	0.014	0.129
	Cervical cancer	0.9158 (0.8208-1.0218)	0.1156	0.080	0.559
	Breast cancer	**0.9452 (0.9096-0.9822)**	**0.0040**	0.083	0.871
	Skin cancer	1.1181 (0.9196-1.3594)	0.2629	0.197	0.945
Ulcerative colitis	Gastric cancer	**1.1240 (1.0624-1.1891)**	**4.7359×10^–5^**	0.079	0.058
	Esophageal cancer	1.0073 (0.9208-1.1020)	0.8732	0.693	0.284
	Bile duct cancer	**1.3107 (1.0983-1.5641)**	**0.0027**	0.800	0.360
	Hepatocellular carcinoma	**1.2365 (1.1235-1.3608)**	**1.4007×10^–5^**	0.139	0.293
	Pancreatic cancer	0.9836 (0.8337-1.1605)	0.8448	0.332	0.660
	Lung cancer	**1.1313 (1.0280-1.2449)**	**0.0116**	0.054	0.819
	Cervical cancer	**1.3941 (1.1708-1.6599)**	**0.0002**	0.115	0.871
	Breast cancer	1.0500 (0.9964-1.1064)	0.0678	0.458	0.179
	Skin cancer	1.1761 (0.8834-1.5659)	0.2666	0.293	0.098

The results show the ORs and CIs after excluding outliers. The bold values mean P-_IVW_<0.05.

## 4 Discussion

There is evidence from many studies that patients with CD and UC are at an increased risk of developing intestinal cancer (6, 7, 44, 45). Interestingly, a growing body of research evidence indicates that these patients are at significantly elevated risk of extraintestinal cancers (46). Our MR study may provide some new evidence on the extraintestinal cancer risk in patients with CD and UC.

During analyses of causal effects between CD and extraintestinal cancers, we discovered that the incidence of pancreatic cancer in patients with CD is 1.10 times higher in the European population and 1.19 times higher in the East Asian population compared to individuals without CD. Similarly, Yu’s MR study showed a causal effect between CD and pancreatic cancer risk (OR: 1.111; 95% CI: 1.015-1.213) in Europeans (47). Two studies, one involving Scandinavians and the other Koreans, also consistently reported that CD increases the risk of pancreatic cancer (48, 49). A previous study found that IL-18 played a key role in the pathogenesis of both CD and pancreatic cancer through a common pathogenic pathway, affecting the immune response and tumor microenvironment by activating immune cells (50). As for breast cancer, our MR analysis showed that the risk was slightly increased in CD patients in Europe (OR: 1.0208; 95% CI: 1.0079-1.0339; P=0.0015). Pellino et al. reported that CD was an independent risk factor for developing breast cancer (OR: 2.76; 95% CI: 1.2-6.2; P=0.017) (51). Existing studies indicated that CD and breast cancer may share common molecular mechanisms (52–54). However, we found that the risk of breast cancer was reduced by 5.48% in CD patients (OR: 0.9452; 95% CI: 0.9096-0.9822) in East Asia. Although previous cohort studies have shown a decreased risk of breast cancer in patients with CD (55, 56), future research is needed to explore the underlying mechanisms that influence cancer risk variation across diverse populations. The genetic profiles of CD patients may differ between European and East Asian populations, leading to varied susceptibility to developing breast cancer.

In our MR study, the effect size of CD on the risk of breast cancer in Europeans was relatively small (OR=1.0208), suggesting that the elevated risk is just modest. Furthermore, in East Asians, the risk of breast cancer may even be reduced. Therefore, it is not recommended for CD patients to conduct earlier or more frequent breast cancer screening compared to the general screening programs currently in place.

In addition, the result from the IVW method indicated a potential association between CD and skin cancer in the European population (OR: 1.0267; 95% CI: 1.0062-1.0476). The pathogenesis of the enhanced risk of skin cancer in CD is poorly understood and may be associated with underlying immune dysfunction in CD patients, leading to altered tumor surveillance (14, 57). However, the MR-Egger method showed OR<1, inconsistent with the direction of IVW. Consequently, the causal association between CD and skin cancer cannot be definitively established in this study. More studies are needed to further determine the causality.

When analyzing causal associations between UC and extraintestinal cancers, we found that the risk of cervical cancer in UC patients is 1.11‐fold higher in the European population and 1.39‐fold higher in the East Asian population compared to individuals without UC. Similar results were reported in several previous studies, suggesting that UC increased the risk of cervical cancer and recommending that women with UC receive regular cervical cancer screening (11, 49, 58, 59).

In Europeans, similar to CD, UC only slightly increased the risk of breast cancer (OR: 1.0220; 95% CI: 1.0051-1.0393). In East Asians, we did not find any correlation between UC and breast cancer. Therefore, we also do not recommend UC patients to undergo breast cancer screening earlier or more frequently than the general screening programs. Interestingly, UC was significantly positively associated with gastric cancer in the East Asian population. Nissen et al. conducted two case-control studies and found that UC was more likely than CD to be a risk factor for gastric cancer (60). Since studies on the relationship between UC and the risk of gastric cancer are limited in the Asian population, more relevant research should be conducted in the future (61). For bile duct and hepatocellular cancers, a large number of studies have suggested that IBD is positively related to the risk of hepatobiliary cancers (62–64). However, our MR study only identified the effect of UC in increasing the risk of bile duct and hepatocellular cancers in the East Asian population. Notably, a previous study involving 17,052 patients with IBD reported that the risk of hepatobiliary cancers was elevated only in patients with UC (10). Another study also reported a significant positive association between UC and bile duct cancer (65). This observation may be associated with the fact that up to 5% of UC patients develop primary sclerosing cholangitis (PSC), a condition known to carry a lifetime risk of developing bile duct cancer ranging from 10% to 15% (66, 67). As for the potential mechanism of the association between IBD and hepatocellular carcinoma, it may be related to the sharing of immune-related biomarkers (68).

Interestingly, our MR study found a potential positive association between UC and lung cancer risk in the East Asian population. A recent study has demonstrated that the lungs and colon can jointly regulate inflammation and immunity through the lung-gut axis, particularly via the transport of gut microbiota and metabolites (69). This provides a possible mechanism for the correlation between UC and lung cancer. Regrettably, two meta-analyses reported an increased risk of lung cancer in CD, but not in UC (10, 70). Although traditional observational studies can offer some initial insight into the association between IBD and cancer, their findings may be influenced by confounders (71, 72). Moreover, our findings diverged from previous observational studies, potentially attributed to the limited sample size of the GWAS dataset.

Our MR study revealed some differences in the risk of developing extraintestinal cancer in CD and UC between European and East Asian populations. In the European population, we found that both CD and UC slightly increased the risk of breast cancer. However, these findings were not found in East Asians. In addition, we found varying degrees of associations between UC and gastric cancer, bile duct cancer, hepatocellular carcinoma, and lung cancer in the East Asian population, but not in the European population. The reasons for these differences in causality remain unclear. It has been reported that there are significant differences in the phenotypes of IBD between Western and Eastern populations (73). Different ethnic groups may have unique genetic markers and susceptibility genes that influence complex genetic diseases (74). These genetic variants can lead to differences in cancer risk among diverse populations.

The strengths of our study lie in the direct assessment of the causal effects of CD and UC on the risk of extraintestinal cancer using the MR method. This approach allows us to avoid the interference of confounding factors in traditional observational studies. Furthermore, our findings provide new evidence for site-specific cancer screening and intervention in patients with CD and UC.

Nevertheless, this study has several limitations. First, the GWAS data for this study are derived from European and East Asian populations, which limits the application of our findings to other populations. Hence, future studies are required to verify the applicability of our results to different populations. Second, we cannot stratify the analysis by sex due to the lack of sex-specific GWAS data. Finally, the MR study can only analyze the causality and cannot explain the specific biological pathways. Further research is necessary to investigate the mechanisms behind the associations of CD and UC with the risk of extraintestinal cancer.

## 5 Conclusion

In summary, based on MR analyses and large-scale GWAS data, our study indicated that genetically predicted CD may be a risk factor for pancreatic and breast cancers in the European population, and for pancreatic cancer in the East Asian population. Regarding UC, it may be a risk factor for cervical and breast cancers in Europeans, and for gastric, bile duct, hepatocellular, lung, and cervical cancers in East Asians. Therefore, patients with CD and UC need to emphasize screening and prevention of site-specific extraintestinal cancers.

## Data availability statement

In this study, all GWAS data were extracted from the IEU Open GWAS project (https://gwas.mrcieu.ac.uk/).

## Author contributions

CY: Conceptualization, Investigation, Methodology, Writing – original draft. JX: Data curation, Writing – original draft. SX: Data curation, Writing – review & editing. LT: Writing – review & editing. QH: Writing – review & editing. XZ: Writing – review & editing. YH: Writing – review & editing. TY: Formal analysis, Writing – original draft. ZS: Funding acquisition, Supervision, Validation, Writing – review & editing. 
