# Supplementary material for: Exploring genetic associations of Crohn’s disease and ulcerative colitis with extraintestinal cancers in European and East Asian populations
Source: Front Immunol. 2024 Feb 8;15:1339207. doi: 10.3389/fimmu.2024.1339207 (PMC10885353; doi:10.3389/fimmu.2024.1339207)
Supplement: Supplementary file 1 [file DataSheet_1.zip › Supplementary tables and figures/Supplementary Figures S1 and S2.docx]

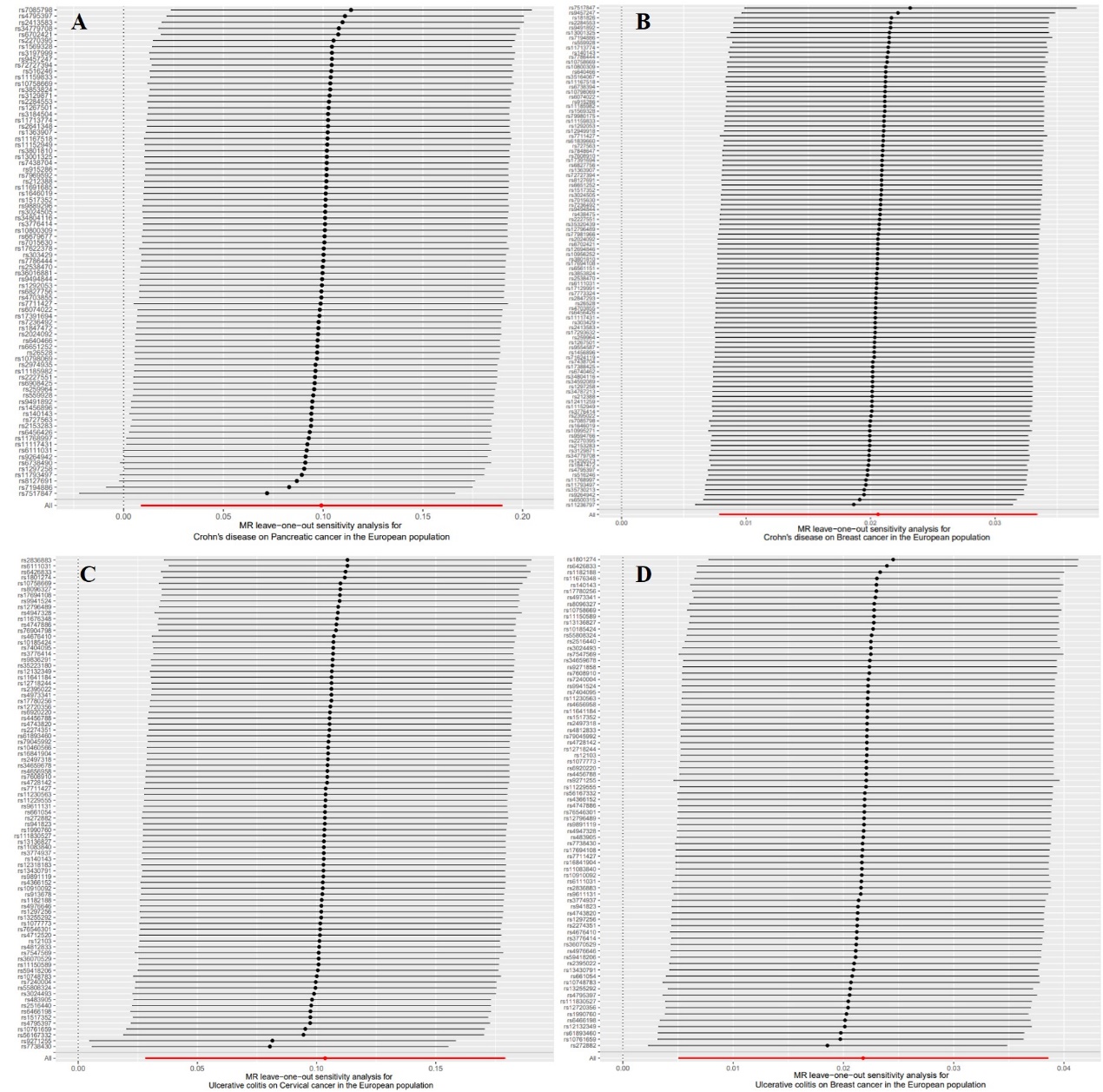


**Figure S1**

The positive results of Leave-one-out analyses in the European population (after removing outliers) (A) between Crohn’s disease and pancreatic cancer (B) between Crohn’s disease and breast cancer (C) between ulcerative colitis and cervical cancer (D) between ulcerative colitis and breast cancer.


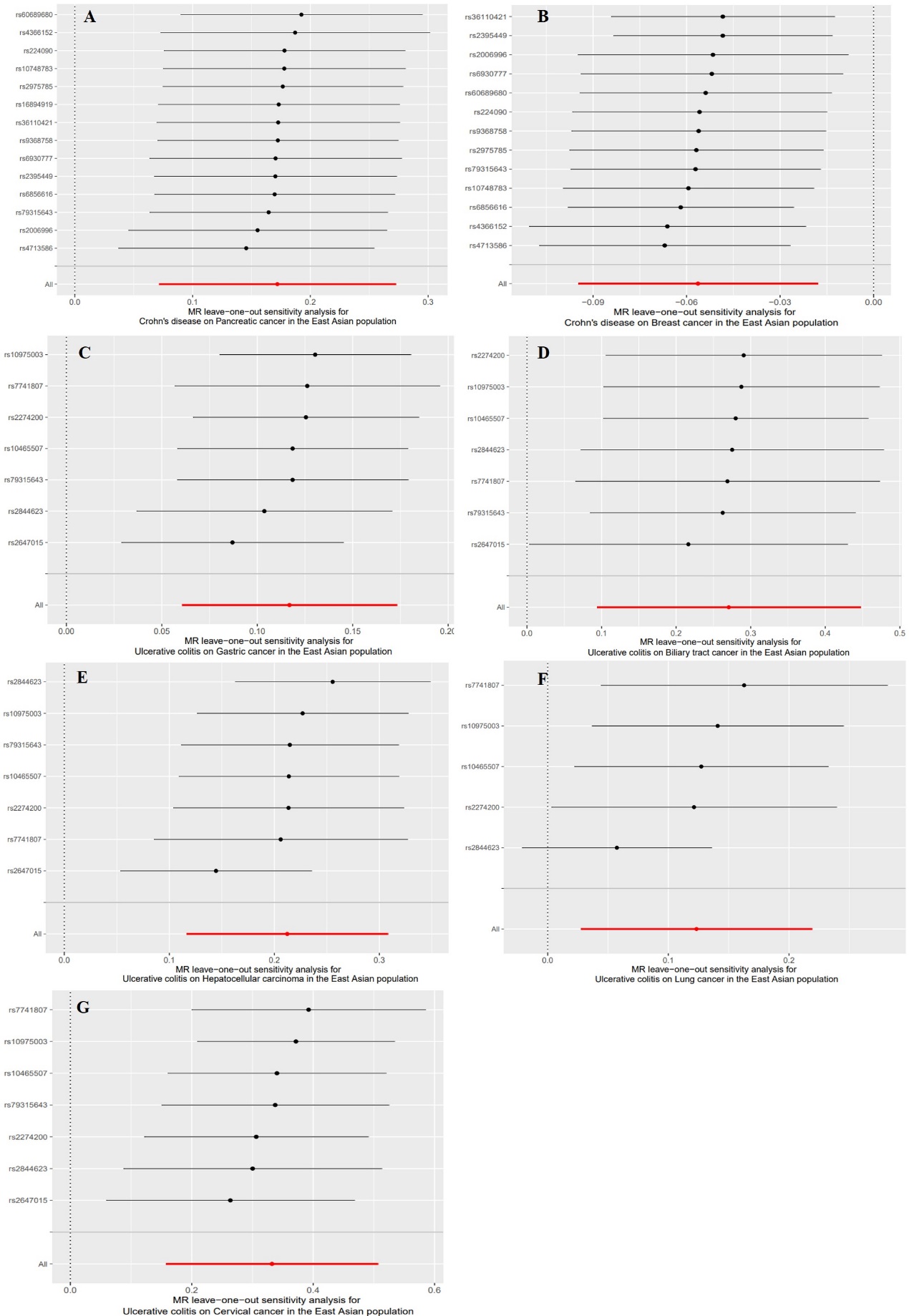


**Figure S2**

The positive results of Leave-one-out analyses in the East Asian population (after removing outliers) (A) between Crohn’s disease and pancreatic cancer (B) between Crohn’s disease and breast cancer (C) between ulcerative colitis and gastric cancer (D) between ulcerative colitis and bile duct cancer (E) between ulcerative colitis and hepatocellular carcinoma (F) between ulcerative colitis and lung cancer (G) between ulcerative colitis and cervical cancer.
